# A case report of primary adrenal lymphoma: A rare but aggressive and invasive disease

**DOI:** 10.1097/MD.0000000000020938

**Published:** 2020-07-10

**Authors:** Yunyun Yang, Wei Xie, Yan Ren, Haoming Tian, Tao Chen

**Affiliations:** a Department of Endocrinology and Metabolism, Adrenal Center; b Department of Radiology, West China Hospital of Sichuan University, Chengdu, Sichuan, China.

**Keywords:** diffuse large B-cell lymphoma, double-expressor lymphoma, primary adrenal lymphoma

## Abstract

**Rationale:**

Primary adrenal lymphoma (PAL) is an extremely rare and highly invasive malignant disease. Imaging examination usually shows bilateral adrenal involvement with large tumor masses and local infiltration. However, it is unclear how lymphoma dynamically develops into huge tumor masses in the adrenal glands. The overall survival rate of PAL is generally poor, and the underlying mechanism might be related to prooncogenic mutation but not fully elucidated.

**Patient concerns:**

A 52-year-old woman complaining of a large mass in the left adrenal region for 1 month was admitted to our department.

**Diagnosis:**

Computed tomography firstly showed a huge mass (8.9 × 7.5 cm) in the left adrenal gland and diffusely enlarged right adrenal gland. A month later, the mass in the left adrenal gland further enlarged (9.5x7.5 cm) with infiltration of the left renal artery and retroperitoneal lymphadenopathy, and the right adrenal gland rapidly progressed into a huge mass (8.0x4.7 cm). Additionally, her chest computed tomography revealed mediastinal and bilateral hilar lymphadenopathy. Then an adrenal biopsy confirmed the diagnosis of diffuse large B-cell lymphoma, nongerminal center B-cell type, stage IV by Ann Arbor staging system. Immunohistochemistry showed positivity for Ki-67 (approximately 90%), BCL2 (approximately 80%) and MYC (approximately 70%) double-expressor lymphoma.

**Interventions:**

The patient's condition progressed rapidly, there was no opportunity to use pathology-based chemotherapy. Dexamethasone was given intravenously by thoracic and intraperitoneal injection; antibiotics and supporting treatment were also given.

**Outcomes:**

The patient's condition progressed rapidly, with the development of malignant chest and abdominal cavity fluid and lung infection, and eventually developed septic shock and respiratory failure. She responded poorly to treatment regimens, and eventually died 8 days after the diagnosis of PAL.

**Lessons:**

PAL grows progressively throughout the adrenal glands, high Ki-67 positivity and BCL2/ MYC co-expression predict rapid progress and poor prognosis.

## 1 Introduction

Adrenal lymphoma consists of primary and secondary adrenal lymphoma. Secondary adrenal lymphoma accounts for approximately 4% to 5% of all non-Hodgkin lymphoma cases, and primary adrenal lymphoma (PAL) is extremely rare, accounting for only approximately 1% of non-Hodgkin lymphoma cases. PAL is based on a histologically confirmed lymphoma involving 1 or 2 adrenal glands, with no previous history of lymphoma, and if other tissues or lymph nodes are involved in addition to the adrenal glands, adrenal lesions must be significantly dominant.^[1–3]^

PAL tends to affect elderly males aged 60 to 70 years old with a male-to-female ratio of approximately 1.8:1, usually involving bilateral adrenal glands (70%), with an average diameter of 8 cm.^[1]^ Most patients with PAL show adrenal insufficiency, B symptoms (fever, night sweats, weight loss), etc, and most patients can be accompanied by elevated lactate dehydrogenase (LDH) and Epstein–Barr virus (EBV) positivity.^[1,4]^

In the imaging examination, PAL mostly presents as bilateral or unilateral large adrenal masses without the appearance of normal adrenal tissue. However, how PAL develops from the adrenal gland is still unclear. Studies suggest that the preservation of the adrenal contour strongly indicates the diagnosis of PAL, indicating that abnormal lymphocytes were growing in the way of diffuse infiltration in the adrenal gland.^[5,6]^ However, there is little imaging evidence of dynamic growth processes at different times in 1 PAL patient.^[7,8]^

Patients with PAL usually have a poor prognosis.^[1,2,9]^ Recently, studies have suggested that BCL-2 and MYC co-expression predict poor prognosis in patients with diffuse large B-cell lymphoma (DLBCL), but there are few reports on BCL-2 and MYC co-expression in patients with PAL.^[10–12]^

To understand PAL better, this study reported a case of PAL with bilateral adrenal involvement, which featured right adrenal gland lesions progressing from diffuse enlargement into a large mass and retroperitoneal lymphadenopathy in 1 month and BCL-2/MYC double-expressor lymphoma. The patient's condition rapidly deteriorated and she eventually died before she could take pathology-guided chemotherapy.

## 2 Case report

**Figure 1:**
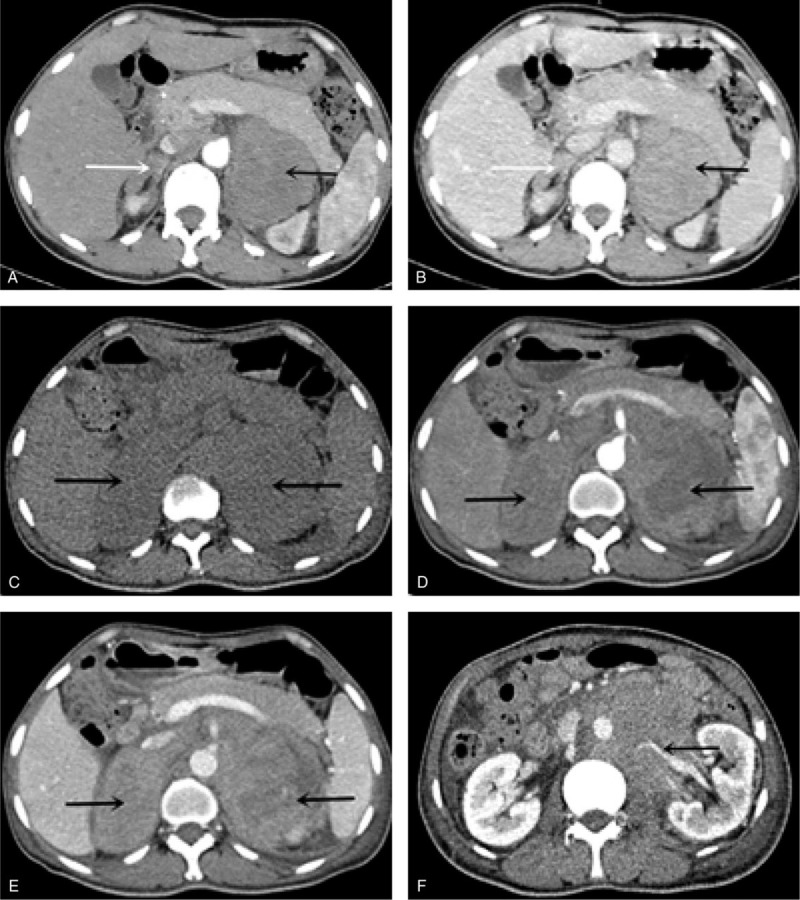
Fast-growing image features of the patient with Primary adrenal lymphoma: compared the first abdominal CT scan (A-B arterial and parenchymal phase) with the second adrenal CT scan (C-E unenhanced, arterial and parenchymal phase), it demonstrated further enlargement of bilateral adrenal masses along with visible bleeding necrosis, local infiltration of the renal artery and retroperitoneal lymphadenopathy.

The patient was a 52-year-old woman who underwent computed tomography (CT) examination due to abdominal pain. A large mass in the left adrenal region and diffused enlargement but intact contour of the right adrenal gland was found (Fig. 1). The patient had been diagnosed with pulmonary tuberculosis 28 years ago and cured after 6 months of treatment. She had denied a medical history of infectious diseases such as hepatitis and HIV, as well as autoimmune diseases. Physical examination showed the following for the patient: heart rate 106 beats/minute, blood pressure 102/71 mm Hg, no hyperpigmentation, no touchable lymph nodes, a painfulness mass in the left abdomen, untouchable liver, and spleen under the ridges of the ribs.

As shown in Table 1, the patient presented with increased LDH and C-reactive protein (CRP); elevated tumor markers (CA-125, NSE); positivity for plasma EBV-DNA and CMV-DNA; negativity for HIV, hepatitis C and hepatitis B antigen test; and a negative tuberculosis-interferon gamma release assay (TB-IGRA) test. Plasma total cortisol (8 and 24 o’clock) and 24-hour urine free cortisol levels were normal. The ACTH level was slightly elevated at the initial measurement and returned to normal at the next-day remeasurement. Her serum catecholamines were slightly elevated, and plasma renin activity, angiotensin-II and plasma aldosterone concentration were normal.

**Table 1:**
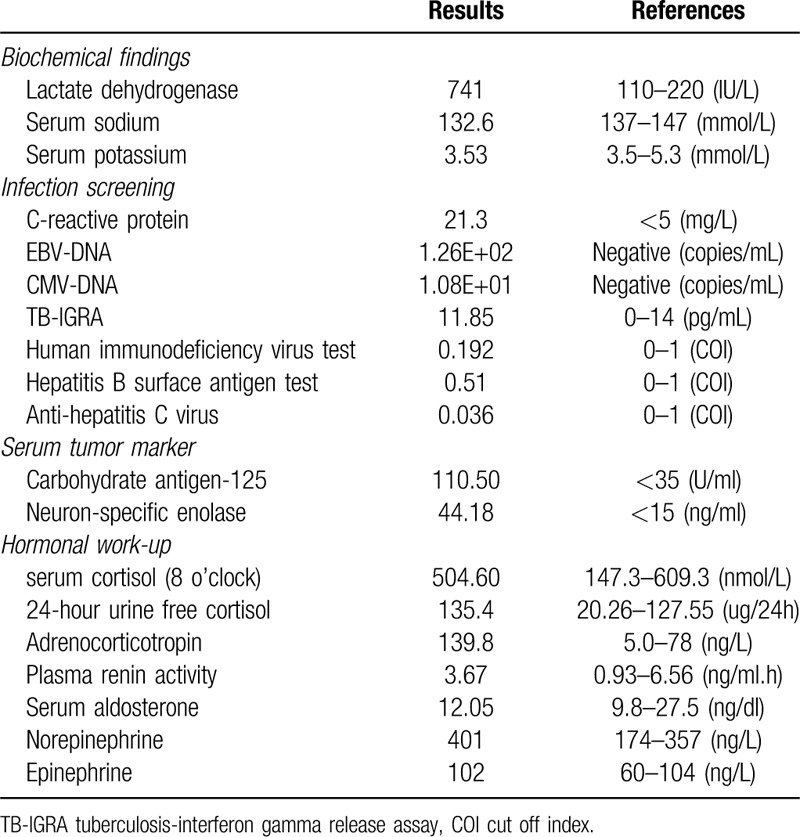
Laboratory findings.

Abdominal CT at first examination revealed a large mass (8.9 × 7.5 cm) in the left adrenal region and diffusely enlarged right adrenal gland with intact contour. The patient had not accepted any treatment due to lack of diagnosis. Approximately 1 month later, repeated CT showed that the mass on the left side had further enlarged (9.5 × 7.5 cm) with bleeding necrosis, local infiltration of the renal artery and retroperitoneal lymph nodes. More importantly, the contour of the right adrenal gland disappeared and was replaced by a large mass (8.0 × 4.7 cm) (Fig. 1). Additionally, chest CT revealed mediastinal and bilateral hilar lymphadenopathy.

The patient accepted ultrasonography-guided biopsy of the left adrenal mass. Pathological studies showed that the molecular markers of tumor cells changed as follows: CD20 (+), CD79a (+), CD3ε (-), CD10 (-), BCL6 (+), Mum-1 (+), CyclinD1 (-), CD30 (-), CD5 (-), BLC2 (+, approximately 80%), MYC (+, approximately 70%), P53 (+), NF-KB (plasma +), Ki-67/MIB-1 (+, approximately 90%); EBER1/2 (-) by in situ hybridization, IGH and IGK clone amplification peaks by Gene Rearrangement (PCR-GENESCAN). The patient was finally diagnosed as diffuse large B-cell lymphoma, non-GCB type by Hans classification, stage IV by Ann Arbor staging system.

During the period of waiting for pathological results, the patient's condition, such as malignant ascites, pleural effusion, and pulmonary infection, were gradually aggravated, and she developed septic shock and type I respiratory failure. Dexamethasone was given intravenously and through thoracic and intraperitoneal injection; antibiotics and supporting treatment were also administered. However, the patient responded poorly to these treatment regimens and died 8 days after confirmation of diagnosis. Because the patient's condition progressed rapidly, there was no opportunity to use pathology-based chemotherapy.

## 3 Discussion

The case reported in this study was a 52-year middle-aged woman, mainly manifesting with abdominal pain, without B symptoms and hyperpigmentation. Laboratory tests showed increased levels of LDH and CRP and positive plasma EBV-DNA. The patient had a history of tuberculosis (TB) but had been cured 28 years ago, and there was no evidence of active TB, no specific infections such as HIV and hepatitis, or autoimmune diseases. The repeated CT examination showed bilateral adrenal lesions. The clinical and imaging characteristics of the case were consistent with the reported PAL cases.^[1,3,9]^

Other causes of bilateral adrenal masses include metastatic diseases (such as lung cancer, lymphoma, melanoma, renal cancer and ovarian cancer), pheochromocytoma, adrenocortical carcinoma, bilateral macronodular hyperplasia, congenital adrenal hyperplasia, myelolipoma, functioning adenomas, infiltrative causes (sarcoidosis and amyloidosis), and infections (tuberculosis, cytoplasm bacteria and cryptococcosis).^[4,13]^ If PAL was suspected according to clinical features (bilateral adrenal lesions in imaging, B symptoms, elevated LDH, decreased HDL, EBV positivity, etc), CT, or ultrasonography-guided biopsy should be performed once pheochromocytoma was excluded. The final diagnosis of PAL is based on the biopsy results of the adrenal mass, and an adrenal biopsy of this patient confirmed the diagnosis of DLBCL.

Surprisingly, although the repeated CT scan indicated that this patient's bilateral adrenal glands were affected with complete loss of normal adrenal contour, she did not develop symptoms or signs of adrenal insufficiency (AI). Laboratory tests showed that her serum sodium and potassium levels were normal, and plasma total cortisol and ACTH did not indicate AI (plasma total cortisol 504.6 nmol/L and 597.20 nmol/L, ACTH 139.8 ng/L and 27.09 ng/L). Several studies showed that 61% of patients with PAL could develop AI.^[1]^ The risk factors included older age, bilateral involvement, and previous autoimmune adrenalitis.^[1]^ However, other studies proposed that AI caused by malignant tumors required destruction of approximately 90% of the adrenal glands, and the correlation between tumor size and AI was weak.^[1,6]^ The present case was relatively young (52 years vs 62–68 years in published studies) and with a short duration (approximately 2 months) without a history of autoimmune diseases, all of which could be the reason for the unimpaired adrenocortical function.

Another interesting feature of this case was the dynamic change in the right adrenal lesions. At the initial CT examination, the patient presented diffusely enlarged right adrenal glands with intact contours. Only 1 month later, repeated CT scan showed that the typical shape of the right adrenal gland disappeared and was replaced by a large mass (8.0x4.7 cm). This finding provided direct proof that lymphoma cells spread in the adrenal gland firstly and diffusely grow subsequently, leading to adrenal gland enlargement, then destruction of the normal structure, and finally merging into a huge mass. Other studies also reported the rapid growth of PAL lesions, but no similar study described the growth patterns of PAL.^[7,8]^ Several studies reported that the rapid increase in PAL tumors might be due to the overexpression of Ki-67, an important indicator that reflects tumor cell proliferation.^[4,6]^ In this case, Ki-67 was highly positive (approximately 90%).

In addition, the patient's IHC test revealed co-expression of BCL-2 and MYC in lymphoma tissues, which indicated double-expressor lymphoma (DEL). DEL is defined as positivity of both MYC and BCL2 in lymphoma tissues by IHC (Cut-off values: 40–50% for MYC, 50–70% for BCL2). Studies have shown that most of DLBCL (not PAL) patients with DEL usually had an aggressive clinical course characterized by poor prognosis, advanced stage (III, IV), more extranodal involvement (including bone marrow, the central nervous system, lung, liver etc), high serum lactate dehydrogenase levels, and an intermediate to high international prognostic index(IPI) score.^[14]^

PAL is highly aggressive and with poor prognosis. A systematic review of PAL indicated that the 3-, 6- and 12-month survival rates of PAL were 67%, 46%, and 20%.^[1]^ Other studies reported that the 2-year, 5-year overall survival (OS) and progression-free survival (PFS) rates of PAL were 61.6% and 49.9%, 52.5% and 53.2%, respectively^[2,9]^ Another study reported that the estimated 5-year and 10-year OS rates of PAL were 19.17% and 3.33%, respectively.^[15]^ Several factors such as older age, bilateral lesions, adrenal insufficiency, B-symptoms, large tumor burden, elevated LDH, central nervous system (CNS) relapse and non-GCB type were identified as adverse prognostic factors for the OS and PFS in patients with PAL, although there is no evidence from randomized clinical trials.^[13,15]^ However, compared to patients without treatment, PAL patients underwent chemotherapy were positive prognostic factors for predicting OS (HR = 0.322, 95% CI 0.198–0.975, *P* < .001).^[15]^ Other predictors for good prognosis include patient's good response to chemotherapy and chemotherapy tolerance.^[4,13]^

Chemotherapy with R-CHOP (rituximab, cyclophosphamide, doxorubicin, vincristine, and prednisone) is the widely recognized strategy for PAL. Studies have shown that compared to traditional CHOP regimens, R-CHOP regimens have higher complete response (76% vs 56%, *P* < .005) and higher 2-year OS and PFS rates (57% vs 38%, *P* < .001; 70% vs 57% *P* = .007, respectively).^[16]^ Therefore, timely diagnosis and treatment with R-CHOP is the key to improving the prognosis of PAL. Regretfully, this patient's condition progressed rapidly, and she eventually died of infectious shock and type I respiratory failure before she could accept pathology-based chemotherapy.

## 4 Conclusions

In summary, this study demonstrates that the growth pattern of PAL is diffuse infiltrating across the adrenal gland, from diffuse enlargement to a merged adrenal mass. Some patients with PAL could preserve partial adrenocortical function despite a high frequency of adrenal insufficiency in PAL. High positivity for Ki-67 and co-expression of BCL2 and MYC account for aggressive growth and increased mortality.

## Author contributions

TC conceived the study and participated in its design and coordination.

YYY collected the clinical information of the participants and drafted the manuscript.

YR collected the clinical information of the participants.

HMT edited the manuscript.

All authors read and approved the final manuscript.
